# Assessment of factors that influence timely administration of initial antibiotic dose using collaborative process mapping at a referral hospital in Malawi: a case study of pneumonia patients

**DOI:** 10.1186/s12879-018-3620-9

**Published:** 2018-12-27

**Authors:** Chimwemwe Tusekile Mula, Lyn Middleton, Nicola Human, Christine Varga

**Affiliations:** 1 Department of Clinical Nursing, Kamuzu College of Nursing, University of Malawi, Blantyre, Malawi; 2 Department of Pharmacy, School of Health Sciences, University of KwaZulu-Natal, KwaZulu-Natal, South Africa; 3 Ohio State University Wexner Medical Center, Columbus, USA

**Keywords:** Antimicrobial resistance, Antimicrobial stewardship, Dose optimization, Timely antibiotic initiation, Pneumonia, Process mapping, Health care system, Bottlenecks, Multidisciplinary team

## Abstract

**Background:**

Timely initiation of antibiotics within one hour of prescription is one of the recommended antibiotic stewardship interventions when managing patients with pneumonia in the emergency department. Effective implementation of this intervention depends on effective communication, a well-established coordination process and availability of resources. Understanding what may influence this aspect of care by using process mapping is an important component when planning for improvement interventions. The aim of the study was to identify factors that influence antibiotic initiation following prescription in the Adult Emergency and Trauma Centre of the largest referral hospital in Malawi.

**Methods:**

We conducted a prospective observational case study using process mapping of two purposively selected adult pneumonia patients. One of the investigators CM observed the patient from the time of arrival at the triage area to the time he/she received initial dose of antibiotics. With purposively selected members of the clinical team; we used simple questions to analyze the map and identified facilitators, barriers and potential areas for improvement.

**Results:**

Both patients did not receive the first dose of antibiotic within one hour of prescription. Despite the situation being less than ideal, potential facilitators to timely antibiotic initiation were: prompt assessment and triaging; availability of different expertise, timely first review by the clinician; and blood culture collected prior to antibiotic initiation. Barriers were: long waits, lack of communication/coordinated care and competency gap. Improvements are needed in communication, multidisciplinary teamwork, education and leadership/supervision.

**Conclusion:**

Process mapping can have a significant impact in unveiling the system-related factors that influence timely initiation of antibiotics. The mapping exercise brought together stakeholders to evaluate and identify the facilitators and barriers. Recommendations here focused on improving communication, multidisciplinary team culture such as teamwork, good leadership and continuing professional development.

**Electronic supplementary material:**

The online version of this article (10.1186/s12879-018-3620-9) contains supplementary material, which is available to authorized users.

## Background

The emergence of drug-resistant organisms is a global health crisis [1]. In response to this threat, antimicrobial stewardship programs (ASPs) have been formed to maximize the benefits of antibiotics while minimizing their unintended consequences [2]. One of the strategies of ASPs is optimizing the dose and timing of antimicrobial agents based on patient and disease [2].

Evidence shows that timely initiation of antibiotics within 4 hours of patient arrival in the emergency department is associated with reduced mortality in patients with community acquired pneumonia [3]. Similarly, timely antibiotic initiation within 3 hours of triage in patients with sepsis is associated with improved outcome [4]. Evidence also demonstrates that rapid initiation of antibiotics within 1 hour of prescription, preferably before a patient leaves the emergency department, is one of the recommended practices for containing pneumonia [5, 6]. In summary, the acceptable time for antibiotic initiation, based on literature, is three to 4 hours after the patient arrives in the emergency department, and within 1 hour of antibiotic prescription.

However, multiple barriers affect these recommendations resulting in delayed administration of the initial dose of antibiotics [7]. In the emergency department, some patients require appropriate attention amidst assessment by multiple providers [8]. In addition, barriers to implementation such as poor collaboration between departments exist [9], overcrowding in the emergency department with high numbers of patients being admitted also affects timely antibiotic administration [10]. This is even more challenging with acute conditions like pneumonia where treatment is largely based on timely prescription of antibiotic therapy [11].

Pneumonia is a common, costly, and potentially fatal illness [12]. At one referral hospital in Malawi, pneumonia was found to be one of the most common admitting diagnoses [13]. In this study setting, antibiotic initiation within 1 hour of prescription is the strategy in practice for patients triaged as priority. The effectiveness of timely antibiotic initiation is determined by timely assessment and triaging; examination by the emergency department physician and then the medical physician; and timely collection of microbiology sample prior to administration of the initial dose. These steps are similar to recommended guidelines in sepsis management [14]. Therefore, as part of an effort to improve timely antibiotic initiation, process mapping was undertaken to identify factors in the environment that affect this care process.

A process map is a diagram of the sequence of major action steps [15] and decisions in a work setting [16]. It is used to track the flow of information, materials, documents, stakeholders and their impact on the process of patient care by clarifying tasks and decisions of actors [17]. It helps to delineate bottlenecks, inefficiencies and redundant steps [18], which can be improved later [19].

Process mapping has been used in various conditions, such as chronic obstructive pulmonary disease [20]; service contexts, such as outpatient attendance [19]; to understand care communication patterns [15]; and to investigate aspects of care requiring improvements for patients coming from other geographical locations such as rural areas [21]. Mapping has also been used to describe the interaction between a clinician, other providers and the endoscopy care process [22]. Mapping can be achieved with case note reviews [23], examinations of protocols [24], observations, patient self-reports and multidisciplinary meetings [22]. There is a dearth of literature about process mapping in the Malawian context and especially for pneumonia patients’ antibiotic therapy initiation.

## Methods

### Aim

The aim of the study was to identify factors that influence timely antibiotic initiation following prescription in the Adult Emergency and Trauma Centre (AETC) of a referral hospital in Malawi and determine areas for improvement**.**The study seeks to answer the following research question: How do environmental /system related factors affect timely antibiotic initiation?

### Design

A case study design [25] was used to study the antibiotic initiation process of patients with pneumonia. We conducted a prospective observation to map the patient journey followed by a meeting with the clinical team to analyze the process. The observation involved observing the pathway firsthand [13] p25 and then discussing the map with the clinical team. The role of the first author, CM, was to shadow (follow) [26] the patients (cases) through each step of the process [21, 22] and map the process as it happened, which resulted in multiple steps [27–29]. The scope of the mapping was from patient arrival at the triage area in the AETC to the time the first dose of antibiotic was given. A similar methodology was used by Johnson [15] to demonstrate how process.

mapping illustrates handover practices between ambulatory and inpatient care settings.

### Study population

We used a typical case sampling [30] where we found that pneumonia was the most common medical condition requiring antibiotics in the AETC. In addition, it was observed that timely administration of antibiotics was a challenge.

Therefore, two patient participants, male and female, were recruited with assistance from the nurse in charge. The sample size was adequate because a case study research aims to maximize the lessons learnt, thus one or more cases are considered enough [25]. The inclusion criteria were: clinically diagnosed as having pneumonia, triaged as priority; Which means the patients had a potentially serious but not immediately life-threatening condition and were assigned second priority for treatment and transportation), aged 18 years and above, able to communicate and have had an antibiotic prescribed.

### Ethics and consent to participate

All patient participants gave their informed written consent. Written informed consent for the health workers encountered in the mapping was not necessary due to the observational nature of the study. The research presented no more than a minimal risk of harm to the health workers and written consent could not practically be carried out because we could not predict in advance who would be found in the care process during mapping. This is in line with the National Health Sciences Research committee in Malawi: https://www.nhsrc-mw.com

In lieu of the signed consent form, a written statement regarding the research in form of information sheet was provided detailing how the verbal consent was documented. The form contained statement about the research, that no special time will be required from the participants, description of the process mapping, risks and benefits, subjects’ rights and contact information of the investigator and COMREC chairperson. Ethics committee approval was granted for verbal consent.Prior to data collection, CM conducted meetings with the nurses and doctors in the AETC and nurses in the two medical wards and used the verbal consent information sheet to ensure team involvement in the improvement process [21]. In addition, we sought verbal consent from the health care worker providing care at the time of mapping. Anonymity was observed by using titles.The study was approved by the University of KwaZulu-Natal Human and Social Sciences Research Ethics Committee (Ref. HSS/0445/015D) and the University of Malawi, College of Medicine Research Ethics Committee (Ref. P03/15/1707).

### Data collection

**Fig. 1:**
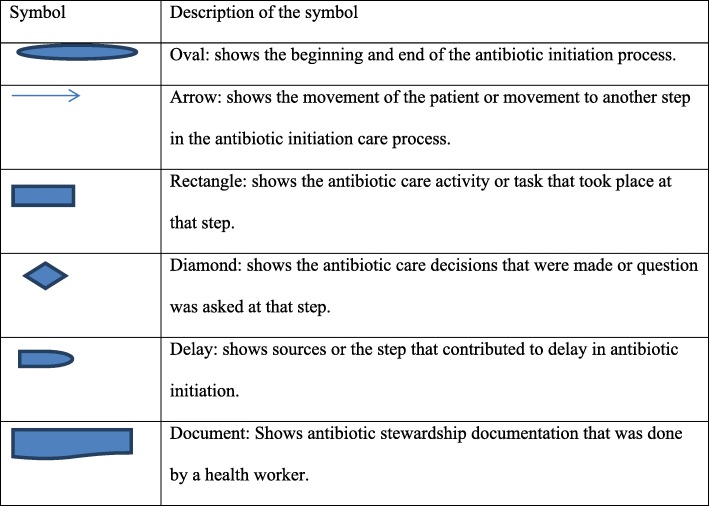
Basic Flowchart Symbols. Is an illustration of basic flow chart symbols that were used to identify specific activities and summarize data. The oval shape shows where the process of antibiotic initiation begun and ended. The arrow shows the direction of patient movement to another step. The rectangle shows the antibiotic care activity or task that took place at that step. The diamond shape shows the antibiotic care decisions that were made at that time. The “D” shape shows the sources or step that contributed to delay in antibiotic initiation. Finally the rectangle with one irregular line shows antibiotic stewardship documentation that was done by a health worker

We developed a mapping guide based on hospital protocols and literature (see Additional file 1). The tool had three sections: patient demographics; physical examination findings; and structure/process indicators. We collected data through direct observations supplemented with case note reviews focusing on the four components proposed by McCarthy [31]: Encounters, Tasks, Actors and Constraints. On both days, CM visited the AETC in the morning and attended the multidisciplinary handover meetings of the previous night’s shift as a method of building a rapport with the health care workers. When a patient arrives in the emergency department, he or she is registered using an electronic record system. Depending on the status and severity of the condition, the patient may be referred to the triage area for assessment. The nurse and CM asked the patient what complaints he or she had, and then performed a preliminary identification of the patient for the mapping. After the clinical diagnosis of pneumonia was made, and antibiotic prescribed, CM confirmed with the patient about the patient’s willingness to participate and obtained their written consent. None of the participants refused participation. The shadower followed the patient, identifying steps, assessing times and recording the flow of relevant information and actions that related to antibiotic interventions at each step of the process. The shadower documented on post-it notes what transpired and then pasted the notes on the flip chart, which was mounted on the wall in the nearby conference room. Basic flow chart symbols (Fig. 1) were used to identify specific activities and summarize data [19, 29]. Each patient journey was treated as a separate case.

### Collaborative analysis of the map

The process mapping involved CM developing an initial understanding of the current process [2], then together with the clinical team, identifying facilitating factors, bottlenecks and solutions to improve the process. The clinical team, that was purposively selected by the first author, comprised the unit matron and deputy registered nurse in charge (coded as 1 and 2 respectively), and male and female doctors (coded as 3 and 4 respectively) from the AETC to participate in analysis of the map. To be more systematic, the team analyzed the map in a structured way using simple questions as guides [15, 16].

### Question guides used to analyze data from the map

- What is happening at each step?
- Where is it taking place?
- Who is involved?
- How many steps does the patient have to complete or how many times is the patient passed from one person to another and are they all necessary?
- How quickly do patients move to second line?
- How long does the whole pathway take?
- What are the successes?
- What are the bottlenecks?
- What opportunities for change are there?

We discussed what actually occurred (versus what stakeholders would like to occur) during various steps in the patient’s journey. We explored the standard practice (what could have happened?).As we discussed, discrepancies started to emerge between what should have happened and what actually happened. We determined the factors (barriers and facilitators) influencing the practice, and located potential areas for improvement based on the clinical team’s experience. The discussion took 1 hour 20 minutes and was tape recorded after obtaining verbal consent from the members.

## Results

Process mapping was conducted at AETC of the largest referral hospital in the urban setting. On average, a total of 5537 adult patient with emergency medical, surgical and trauma conditions are seen per month.

**Fig. 2:**
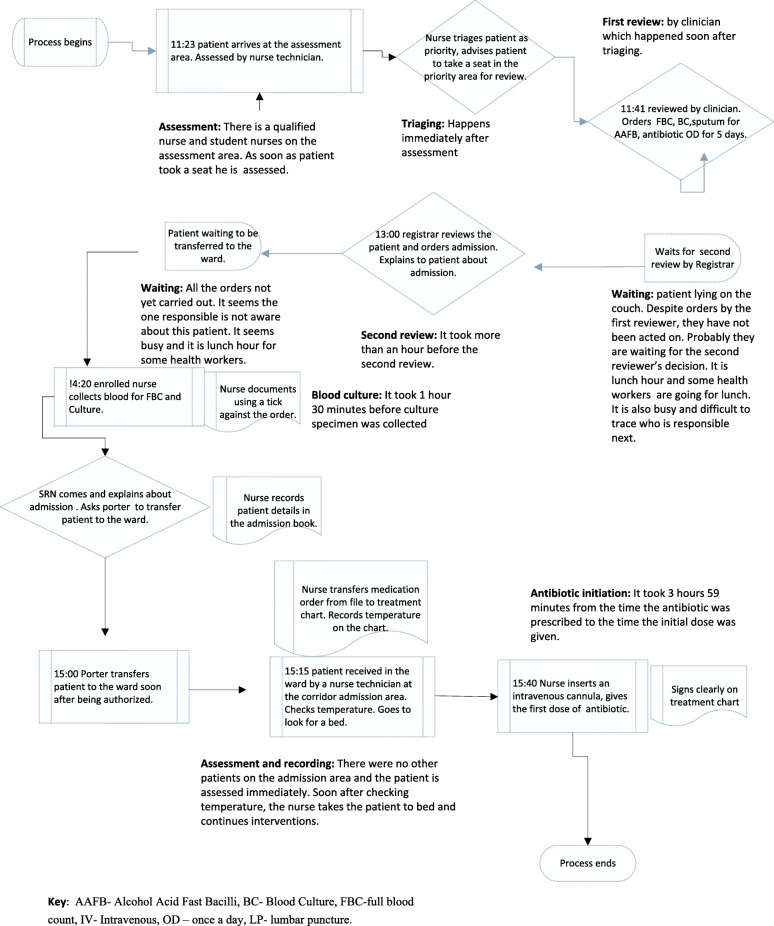
Flowchart of a care pathway in clinical diagnosis and antibiotic initiation of male pneumonia patient. Illustration of the male patient antibiotic initiation data based on direct observation of the process from patient arrival on the assessment area to the time the first dose of antibiotic was given. The observation took 4 h 17 min. Using the relevant symbols; the map shows the actors, actions/ tasks and decisions at each step of the process.Major action steps of the process are highlighted namely assessment, triaging, first review, waiting, second review, blood culture, assessment and recording, and antibiotic initiation. It took 3 h 59 min from the time the antibiotic was prescribed to the time it was given. The potential source of delay in antibiotic initiation was waiting time as shownby the relevant delay symbol. Mapping identified both potential factors that could have influenced timely antibiotic initiation and barriers to timely antibiotic initiation

**Fig. 3:**
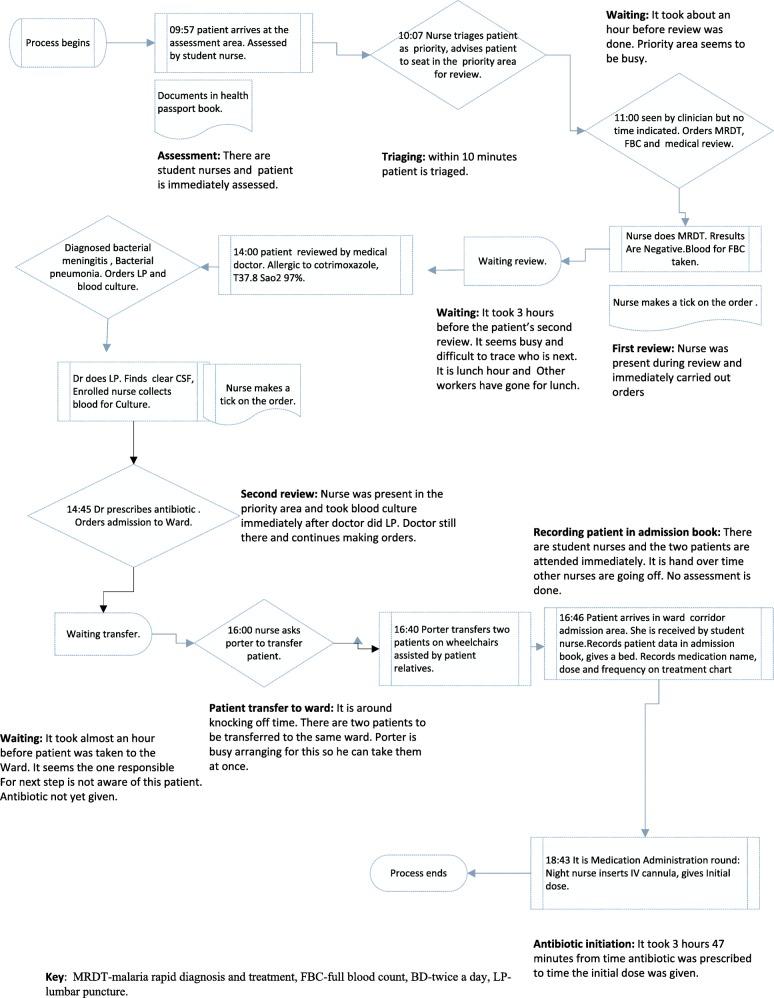
Flowchart of a care pathway in clinical diagnosis and antibiotic initiation of female pneumonia patient. Illustration of the female patient antibiotic initiation data based on direct observation of the process from patient arrival on the assessment area to the time the first dose of antibiotic was given. The observation took 7 h 14 min. Using the relevant symbols; the map shows the actors, actions/ tasks and decisions at each step of the process.Major action steps of the process are highlighted namely assessment, triaging, first review, waiting, recording patient data in admission book and antibiotic initiation. It took 3 h 47 min from the time the antibiotic was prescribed to the time it was given. The potential source of delay in antibiotic initiation was waiting time as shown by the relevant delay symbol. Mapping identified both potential factors that could have influenced timely antibiotic initiation such as having different actors (health workers) and barriers (such as waiting)to timely antibiotic initiation

Following mapping, the researcher wrote the findings as a flowchart diagram for the male and female patients. Figure 2 illustrates the flow chart of a care pathway for the male patient and Fig. 3 for the female patient. The process map shows the actors and actions/decisions at each step. Mapping identified both potential factors that could have influenced timely antibiotic initiation and barriers to timely antibiotic initiation.

### Patient characteristics

The first patient mapped was male. He was followed and observed for 4 hours 17 minutes. The second patient was female. She was followed and observed for 7 hours 14 minutes. These patients had clinical manifestations and vital signs that pointed to a potentially serious respiratory problem and were therefore second priority for treatment and transportation to the ward.

### Potential facilitating factors

The following are responses from the clinical team to a question about the successes of a patient antibiotic management system which could positively influence timely administration of initial dose:

#### Timely assessments and triaging, proper chain of procedures were followed

Firstly, assessment of vital signs namely oxygen saturation, pulse rate and temperature followed by triaging took place immediately upon patients’ arrival. This was facilitated by the consistent presence of a nurse and student nurses in the triage area. In both cases, the triage nurses acted promptly, this is the recommended practice in the department. Timely checking of vital signs that pointed to the infectious process (i.e. temperature) during the triage and admission in the ward is a potential factor that could have facilitated timely antibiotic initiation. One participant during analysis had these positive sentiments about the ward nurse:

*The nurse admitting patient checked temperature, checked orders and was able to see a gap and administered the antibiotic. (Health worker 2)*

The nurse in the ward checked the patient’s temperature (male patient). After identifying a fever and noting that the initial dose of antibiotic was not administered in AETC, she intervened immediately by inserting the cannula and gave the antibiotic. This was a positive action, though the antibiotic was still given after 1 hour of prescription.

#### First review by clinician and carrying out of orders

Secondly, the clinician reviewed the male patient soon after triaging. This was facilitated by the presence of the AETC clinician and a list of guidelines posted on the wall stipulating these rules. However the female patient was reviewed after an hour of triaging because the priority area was busy. Next, the nurses’ presence during clinician review also facilitated timely collection of blood culture specimens in the female patient although the male patient experienced delays. Indeed, nurses are aware that blood cultures should be collected prior to antibiotics.

#### Second review by doctor

In the male patient, second review by doctor happened after 1 h 14 minutes following first review while in female patient it took 2 h.

#### Availability of different actors

All required experts were available during the care process. These were AETC clinicians, Medical clinicians, AETC nurses, blood culture nurses and a porter to potentially facilitate timely antibiotic initiation. In addition, student nurses played a role in assessing and triaging patients in the AETC and receiving patients in the ward. Each actor participated in each step of the care pathway, though their efforts were hampered by other factors as will be seen below. As a result, both patients did not receive antibiotics before leaving the AETC. In this case what needs to improve is the care coordination to ensure that the different actors play their roles optimally hence timely antibiotic initiation would be effected.

### Barriers and challenges

The following extracts capture stakeholders’ responses when asked what the barriers were to timely antibiotic initiation.

#### Incomplete actions attributed to material resource challenges

*This is mainly due to faulty blood pressure machine. (Health worker 2)*

*A lot of them (nurses) do not have watches so they don’t check respirations. (Health worker 3)*

Despite nurses performing timely assessment and triage, it seems they were not able to complete the assessment due to challenges with equipment. For example, in the first action the assessment task is incomplete; both patients did not have their respirations and blood pressure checked even though these are vital parameters when managing a patient with an infectious process.

#### Long waits attributed to staff shortage and hand offs

*Initially, we had a clinician allocated to a cubicle (compartment where each patient is assessed) but now clinicians are no longer allocated according to cubicle because now they are less to share the cubicles. (Health worker 4)*

*A good number of the patients have medical conditions so with one clinician it’s a challenge. (Healthworker 2)*

First, the map shows several steps requiring hand offs between one health worker and another. These are tasks such as assessments, triaging, first and second reviews, investigations, and patient transfer. According to the AETC protocol, the patient is reviewed first by the AETC clinician followed by the senior medical clinician to confirm admission. Thus, it took a long time to access the second clinician. The second clinician would be attending to other patients in the resuscitation area or in the same priority area which has six cubicles and sometimes all cubicles could be full of medical patients waiting for the second review.

#### Challenges with communication/coordinated care

There is minimal direct verbal and written communication especially between nurses and doctors:

*Clinicians (after reviewing first patient) may be catching up with other patients (rushing to see the next patient) off they proceed. So communication in the cubicles from both sides is a problem. Documentation is an issue. They can give the antibiotic but they don’t document. People have repeated the dose because of no documentation. (Health worker 1)*

In our study, we found that there were challenges with direct communication: 1) between the first and second clinicians, 2) between the nurse and the clinician, and 3) among nurses, which resulted in uncoordinated care. Documentation is also a challenge for the nurses.The first clinician may review a patient and write down the orders in the absence of the nurse who might be seeing another patient. As the clinician is trying to work quickly to see other patients, verbal communication does not take place; as a result the nurse is not aware of the orders, which then may not be acted on in time. The AETC department has research nurses responsible for collecting blood cultures (blood culture nurses). After collecting the blood, if the blood culture nurse does not inform the AETC nurse to come and give the antibiotics, this may contribute to the delay. At the same time, when a blood culture is ordered and the blood culture nurse is not present, sometimes the AETC nurse does not collect the specimen and waits for the former.

There were also delays in communication between nurse and porter regarding patient transfer. For example, the male and female patients had to wait for about 40 min and one hour respectively after an admission was ordered, before being transferred to the ward due to poor communication.

#### Coordination of care was another barrier

*During lunch hour, there is a little bit of slow down on activities. Lunch hour is mostly busy, more patients coming, people (health care workers) are tired and others have gone for lunch. (Health worker 1)*

Health care delivery during the lunch hour proved to be ineffective as more patients require assistance, yet fewer health care workers are available. Staffing patterns during lunch hour and shift change handoffs affected the process of care.

#### Competency gap is a barrier to the process

Knowledge gaps, poor attitudes and inexperience were also mentioned as possible barriers influencing delays:

*Attitude and to sustain standards is a challenge, unless policing. Lack of knowledge and low confidence level (referring to nurses). Because even before second clinician reviews patient, the nurse is supposed to carry out the emergency orders. (Health worker 1)*

*Some clinicians clear (review) patients automatically and faster. But it depends; an intern may take longer to see one patient because he has to concentrate as does not have much experience but when with registrar it’s faster. So level and also experience matters. (Health worker 3)*

First, it seems the sustainability of the standards is a challenge as actors need to be closely monitored for them to comply. This demonstrates knowledge, attitude and supervisory gaps. Secondly, doctors’ experience and knowledge affects the process as it takes longer for an inexperienced doctor to complete the same task.

### Areas for improvement

The findings about barriers informed our recommendations for improvement.

#### Team work: Verbal communication/coordination and documentation

*A nurse should be there (allocated) already and carry out orders but if the nurse is not there, the clinician is supposed to communicate after review. (Health Worker1)*

*Both verbal and written communication is an area to improve and collaboration between a clinician and a nurse. This will help to prioritize care. Documentation by ticking against the order after it is carried out (i.e. blood culture) is acceptable considering the volume of work and the urgency in the AETC. (Health Worker3)*

There is a need to improve nurse-doctor communication and documentation. Documentation using a tick (√) symbol only is not enough and the team analysing the data agreed that the time the action is taken should be documented in addition to the tick.

*AETC clinician should communicate with medical clinician. They are supposed to consult, sometimes depends on clinician. (Health Worker 1)*

There is also a need to improve communication amongst clinicians.

*Blood Culture nurse could have communicated with AETC nurse after collection of blood culture. But even Blood Culture nurse can insert cannula and give the antibiotic. Mostly after blood culture they administer antibiotics but documentation is an issue. (Health Worker 2)*

The AETC and Blood Culture nurses also need to improve their communication and coordination of care.

*During patient transfer, nurse is supposed to check again in the file to ensure all is done. Some clever porters may ask if they can take patient or remind nurses. (Health Worker 1)*

Similarly, the nurse and the porter need to improve their communication.

#### Leadership and supervision

*Leadership - we cannot run away from it. Spot checking on how things are going should be done. Lead nurse could be checking the priority patients not yet reviewed to ensure patients are reviewed and orders are carried out. (Health Worker1)*

Another key issue the participants recommended was leadership. There is a need to spot check to ensure care is delivered in a timely manner. This should be the responsibility of the nurse leader who is assigned to an area on each shift in the AETC to ensure supervision takes place.

#### Awareness about importance of antibiotics

*People (health care workers) need to understand the importance of timely antibiotic initiation, there should be emphasis on this just is the case with those patients that require resuscitation. It should be part of Continuing Professional Development (CPD). (Health worker 3)*

*If the porter also had some knowledge, to remind the nurse that they have not given the antibiotic prior to transfer. But the challenge is they just transfer the patient with little knowledge. (Health Worker1)*

It seems there is a need for CPD so that health workers understand the importance of timely antibiotic initiation. Similarly, the porter responsible for transferring the patient should have some basic knowledge about the patient being transferred.

## Discussion

Understanding the process of initiating antibiotics within 1 hour of prescription has implications for the nurses and clinicians to improve practice. Involving stakeholders helped to get a better perspective of the process that was based on their experience. This led to a better interpretation of events [14]. Based on the objective, we identified four themes under the domain ‘potential facilitating factors’; five themes from domain ‘barriers/bottlenecks’; and three themes from the domain ‘areas for improvement’.

Evidence shows that timely assessment facilitates timely antibiotic initiation [32]. However, the results of the mapping show that while there was timely assessment and triage of the patient and that there were different actors in the process as potential facilitating factors to timely antibiotic initiation, waiting time for antibiotic initiation was prolonged. Similarly, adhering to the procedures of timely review and carrying out of microbiology specimen orders as it happened in the male patient could potentially lead to timely identification of the causative organism and timely antibiotic initiation. This is in line with Hatton [33] who states that obtaining appropriate cultures before initiating antimicrobial therapy plays an important role as the chances of identifying the offending microorganism are improved. However this was affected by prolonged waiting times for the female patient.

Antibiotic initiation delays in this study therefore demonstrate poor quality of care. There was variation in the time it took between a male and female patient to get prescribed antibiotic. The male and female patients took 3 hours 59 min and 3 hours 47 min respectively; from prescription to receiving the antibiotic instead of the practice standard of administering it within 1 hour of prescription. In this study, factors in the process contributing to delays were handoffs, staff shortages, control points, poor communication, uncoordinated care and competency gaps.

Excessive handoff is a functional bottleneck as patients are unnecessarily passed from one health worker to another [34]. Staff shortages worsened the delay. Rodriguez et al.*,* [35] assessed processes of care to promote timely antibiotic initiation using surveys and focus group discussions with doctors, pharmacists and radiologists. They identified assessment, shift changes, transportation and communication delays as factors that delayed antibiotic initiation because the steps require handoffs between health care workers. These findings are consistent with the current study.

Valueless steps that delay the process of care are reported. Trebble [22] found valueless steps in the observational mapping study of an endoscopy procedure where five out of the 23 steps added no value to the patient outcome. Similarly, Kainthet al. [36], in a study to determine time taken in the process of management of patient with community acquired pneumonia, found that the longest delay was the process of obtaining a chest x-ray; recommendation was prioritization of chest x-ray by using porters to transfer patients to the radiology department. However, in our study, analysis showed that each step was found to be necessary by the clinical team, except in one step in the female patient. After giving the patient a bed, the nurse was not supposed to wait for the medication rounds to give the initial dose of antibiotic; this waiting for medication round was a valueless step. There is need to prioritize antibiotic initiation irrespective of whether it is medication administration round or not. Nurses need to be educated in this aspect of care and the relevant guidelines and protocols should reflect this.

Related to the handoffs, we noted control points where one health worker needed to provide authorization before the next action [34]. For example, following the review by the AETC clinician, the patient is supposed to be seen by the medical clinician to authorize admission or confirm prescription orders. Similarly, following admission authorization by the medical clinician; the nurse is supposed to authorize the porter to transfer the patient. When the next responsible worker is not aware of the succeeding step or is busy, this delays the process as the following actor cannot act without authorization. This delayed antibiotic initiation further because; for example, if the patient was transferred in time, it would be possible for the ward nurse to give the missed initial dose without further delay.

Similarly, Pinningtonet al., [32] in a quality improvement project found communication barriers that influenced timely treatment in severe sepsis. The communication failures were that staff did not know which nurse/doctor was looking after the patient, there was no communication about the prescribed treatment and staff did not know who was assigned to a specific patient [32]. As a result, interactive multidisciplinary education sessions were identified as important interventions.

Uncoordinated care compounded with staff shortage was another barrier. Spath [37] noted similar findings where there was no correlation between staffing patterns and patient demand and they changed the system for scheduling staff so that working hours reflected predicted levels of demand. Similarly, Watts et al. [38] found that time of the day was a factor affecting timely antibiotic initiation. Therefore, there is a need to increase the staffing levels at the department and consider allocating similar numbers at all times including lunch hours to ensure work is well coordinated.

Competency gap was also evident in this study. There was a lack of awareness of the importance of reduced time to antibiotic initiation as was identified in one study [35]. Though it is suggested that in the AETC, the clinician needs to diagnose rapidly and initiate treatment [39], this may not work well for the less experienced in our study setting, leading to delays.

A common theme throughout discussions about recommendations for improvement has been teamwork. The clinical team analysing data recommended that when a clinician reviews a patient in the absence of a nurse h/she should verbally communicate the orders so the nurse is aware. Clinicians should also consult and communicate whenever a patient on priority is reviewed and requires the review of the second clinician. The same is the case during patient transfer. Before handing over to the porter, the nurse should check the orders to see if the patient has received the antibiotic. Without this care coordination, it is difficult for a multidisciplinary and continuous care to thrive [40]. For example, Downey et al. [41], state that communication between physicians is best done using a formalized process with a consultant contributing in improving the process [42]. There is need for such a formalized process in the current study setting.

The process mapping has shown that synergy is less than optimal, which can lead to poor patient outcomes. The findings revealed implications for engaging stakeholders who made suggestions for improvement. This concurs with Johnson [15] who states that the value of a process mapping exercise is that it clarifies the process for the actors and builds bridges to engage these actors in future improvement work. Similar to the present study, Martin [43] found that engagement of stakeholders was effective in identifying and explaining sources of delay and aspects that could be improved. Based on these findings, improvement interventions have been done collaboratively with stakeholders.

### Strengths and limitations

The map failed to consider the patients’ experiences, which is recommended as critical [20, 31] and did not focus on patient-related factors that could have influenced timely antibiotic initiation as was found in one study [44]. For example, patient presentation may determine the urgency of the actions [45]; this was not considered. In addition, the mapping did not consider diagnostic aspect of the care process that could also affect antibiotic initiation.This quality improvement initiative focused mainly on timing of initial antibiotic therapy. While this is a major issue when it comes to the odds of survival, other factors may pay an important role in the outcomes associated with community-acquired pneumonia [46].

Though lacking this integration, the map offers a guide to a solution on the system improvement by answering critical questions. This is in line with the United Kingdom’s National Health Service (NHS) Institute for Innovation and Improvement [26], which proposes establishing what is currently happening or how efficient the process is, in order to provide a baseline for solutions.

The two cases are too few to generalize how antibiotic initiation is conducted in this particular context. Based on these results, one may have an impression that all patients do not get the initial dose of antibiotic in time. This is not the case as the sample is small and such generalizations do not apply [25]. The results of the analysis were used to develop other interventions that did not involve designing a map for the department. This was beyond the scope of this study. There is a need therefore to design a map that would guide in timely antibiotic initiation based on the findings.

## Conclusion

We have demonstrated that process mapping can have a significant impact in reducing delays in diagnosis and management of pneumonia thus enabling timely administration of initial dose of antibiotic. Evaluating the map indicates positive issues such as timely assessment, triage and the availability of different actors. From this process map analysis, improvements are needed in communication/coordination, teamwork, leadership and awareness about antibiotic stewardship.

## Additional file

Additional file 1: Antibiotic initiation process mapping guide. A data collection tool used during antibiotic initiation process mapping. It shows patient particulars to be collected, process and structure indicators relating to patient antibiotic management. (DOCX 15 kb)

## Abbreviations

AETC: Adult Emergency and Trauma Centre
ASPs: Antimicrobial Stewardship Programs
CPD: Continuing Professional Development
