# Supplementary material for: Assessment of factors that influence timely administration of initial antibiotic dose using collaborative process mapping at a referral hospital in Malawi: a case study of pneumonia patients
Source: BMC Infect Dis. 2018 Dec 27;18:697. doi: 10.1186/s12879-018-3620-9 (PMC6307292; doi:10.1186/s12879-018-3620-9)
Supplement: Supplementary file 1 — Antibiotic initiation process mapping guide. A data collection tool used during antibiotic initiation process mapping. It shows patient particulars to be collected, process and structure indicators relating to patient antibiotic management. (DOCX 15 kb) [file 12879_2018_3620_MOESM1_ESM.docx]

# Additional file 1: Antibiotic initiation process mapping guide

**Start:** Time patient arrives at the assessment area for triaging.

**Patient Particulars (Characteristics):**

Patient Identify number

Age, Sex, Admission Date

Antibiotic prescribed, date and time of prescription

Microbiology specimen/x-ray ordered, date and time

**Process and Structure Indicators in the environment in relation to patient antibiotic management**

Process indicators

Antimicrobial specimen management or any investigations, such as x-rays

- Timely Collection, sending to Laboratory. If no why

Timely administration of antibiotic.

System/processes reflecting:

Multidisciplinary team effort i.e.

- Communications related to antibiotic therapy
- Decision making, level of involvement by nurse
- Leadership
- collaboration

Support system (structure indicators)

- Availability of human and material resources: i.e. staff, guidelines, prescribed antibiotic.

Observe. What does not happen – explore why

*Identify what happens in relation to antibiotic management, how long it takes, any problems, delays, areas of error and confusion, bottlenecks.*

**Finish:** Time Patient receives initial antibiotic dose.
